# Genetic Diversity of *Leptospira* Strains Circulating in Humans and in Free-Ranging Rats Indicates That Rats Are Not Sources of Human Leptospiroses in Hungary 2022–2025

**DOI:** 10.3390/pathogens15070729

**Published:** 2026-07-10

**Authors:** Gabriella Locsmándi, Enikő Kádár-Hürkecz, Zsuzsa Kienle, Katalin Tárnoki-Boross, Krisztina Sima, Tímea Erdősi, László Egyed

**Affiliations:** 1 Department of Bacteriology, Veterinary Diagnostic Directorate National Food Chain Safety Office, 1143 Budapest, Hungary; 2 National Center for Public Health and Pharmacy, 1097 Budapest, Hungary; hurkecz.eniko@nngyk.gov.hu (E.K.-H.);; 3 HUN-REN Veterinary Medical Research Institute, 1143 Budapest, Hungary

**Keywords:** free-ranging rats, lfb1 PCR, Sanger sequencing, *Leptospirae*, humans, Hungary

## Abstract

One hundred and ninety free-ranging rat individuals from dozens of sampling areas (towns, farms, villages) were investigated for *Leptospira* infections. From the renal tissues (kidneys and urinary bladders), DNA was extracted, and the samples were screened by an lfb1-specific PCR assay. The PCR products were sequenced. One hundred and three (54.2%) of the samples proved to be positive. All detected Leptospires belonged to the pathogenic phylogenetic cluster, and only one species was detected: all 103 positive samples belonged to *L. interrogans* lfb1 species group 1. In parallel, 353 human samples (urine, anticoagulated blood, tissues) from 232 patients over the period 2022–2025 submitted with suspected *Leptospira* infections were tested by commercial multiplex real-time PCR kits. Twenty-nine (8%) positive samples were found, which were retested by lfb1-specific PCR. The quality of 20 PCR products was sufficient for sequencing, representing 14 individual patients. Among the 14 positive patients we identified two *Leptospira* species: *L. kirschneri* lfb1 species group 6 in one case and *L. interrogans* in 13 cases (one lfb1 species group 3 imported case, 12 lfb1 species group 2). Comparison of the *lfb1* sequences obtained from rats (lfb1: 1) and human cases (lfb1: 2, 1: 3, 1: 6) indicated that, although rat populations maintain the pathogen in high prevalence, they could not be the sources of the identified human *Leptospira* infections.

## 1. Introduction

Leptospirosis, as a widespread zoonosis across the world, is an emerging public health problem, particularly in large urban centers of the tropics [1,2]. The mortality from severe disease forms, Weil’s disease (>10%) and severe pulmonary hemorrhage syndrome, is high (>50%) even when optimal treatment is provided [3,4], but chronic renal infections and brain hemorrhage also occur [5].

*Leptospirae* are also common animal pathogens. All free-ranging feral, pet [6] and livestock mammal species [7,8] are susceptible to the infection, but as an exception, cats are usually asymptomatic carriers. An immunosuppressive effect is necessary for clinical manifestation in them (Mazotta et al., 2023) [9].

Leptospires are spirochetes, about 0.1 μm in diameter and 6–20 μm in length, and include both saprophytic and pathogenic species comprising the genus *Leptospira*, which belongs to the family Leptospiraceae, order Spirochaetales. *Leptospira* isolates have been grouped many times into saprophytic, intermediate and pathogenic phylogenetic clusters. Nowadays, 31 pathogenic, 21 intermediate and 31 saprophytic *Leptospira* species are grouped into four clades [10]. The saprophytic *L. biflexa* prefers 1–35 °C, while the pathogenic *L. interrogans* lives at 20–37 °C. Pathogenic *Leptospirae* produce proteins that interact by binding to molecules of host cells and the extracellular matrix, the complement system, and the fibrinogen/thrombin system to reduce fibrin clot formation [11]. These abilities (and probably others) enable pathogenic strains to establish persistent infections, mostly in the renal–urinary tract tissues. Hosts are probably infected from the environment, usually from soil and surface waters [12] infected by the urine of *Leptospira* carrier animals or humans. Management practices and the density of animals are important factors in exposure to Leptospires [13].

Most mammalian species are susceptible to *Leptospira* infections but only a few act as efficient reservoirs capable of establishing long-term kidney colonization and excretion of *Leptospirae* in their urine. Many rodent species are considered reservoirs [14,15,16], but livestock species like cattle, sheep, and pigs, and small mammals living around them, like bats [13,17], house mice, black rats and hedgehogs, can all be infected with *Leptospirae* [6,8].

Wild rodent species, especially rats [6,18,19,20], are considered the main and most important reservoirs [16,21]. Rats are particularly important as these species (mostly brown and black rats) live in close proximity to human peridomestic areas and settlements. Rats are highly susceptible to renal *Leptospira* colonialization, which leads to persistent infection and continuous shedding of the bacteria via urine [6,22,23,24]. *L. interrogans* forms a biofilm in the renal tubuli of brown rats, establishing chronic renal colonialization and continuous shedding [23]. Suckling rat pups are also susceptible to *Leptospira* infection [25], but from the age of 23 days they do not show any symptoms of infection. In Europe, *Leptospirae* were found in 7.5% of brown rats and 0.5% of black rats in the Netherlands [26] and in 15% of brown rats in Paris, France [27]. The rat–human infection route is suspected, but the idea is questioned due to contradictory published data: similar serotypes were detected in humans and rats living in the same environment in the Philippines [28], and a study from Boston suggested a link to rats as the source of human *Leptospira* infections [29], but no human leptospirosis was found in a survey study in Canada [30].

*Leptospira interrogans* is the species that is most frequently isolated from rats [15,28] and humans [30], with *L. kirschneri* found less frequently [16,31]. With low prevalence, *L. borgpeterseni* was also reported from free-ranging rodents in China [16], where *Apodemus agrarius* also seemed to be an important host of *Leptospira* infections [15,16]. The reason for the dominance of *L. interrogans* (in contrast to *L. borgpeterseni* and *L. mayottensis*) in samples is probably its capability to establish chronic renal infection in rats; this was proved by experimental infection of brown rats [22], which resulted in continuous shedding of *Leptospirae* in urine to the environment.

Sixty-eight species have been identified under *Leptospira* [32], with eight pathogenic species among them. Later on, these species were further grouped by serological methods on the basis of the O-antigen of the surface lipopolysaccharides into specific serovars [33] (nearly 60 serovars under *L. biflexa* and at least 225 serovars under *L. interrogans* divided in 26 serogroups).

Lfb1 is a putative adhesin molecule of the fibronectin-binding protein family [34], and the DNA sequences of this gene coincide with the classification of pathogenic *Leptospira* strains [35]. With wider polymorphism of lfb1 than the 16S gene, the DNA sequence of this gene allows the identification of groups within a certain *Leptospira* species at the subspecies level [36].

Since data on the prevalence of the pathogenic Leptospires in the most important reservoir species, the rat, are relatively rare in the European temperate-climate zone, we intended to carry out a survey of Leptosira infection at the subspecies level in free-ranging rats and to compare the results to lfb1 sequences identified from Hungarian human leptospirosis cases.

## 2. Materials and Methods

### 2.1. Collection and Preparation of Rat Samples

The processed rat samples were tissues from 190 rats: 179 brown (*Rattus norvegicus*, 94.2%) and 11 black rats (*Rattus rattus*, 5.8%). The animals were fresh (<24 h) poisoned carcasses, collected between 2022 and 2025. One hundred and fourteen rats (60%) were from the capital, Budapest; the remaining 76 (40%) were from 11 counties (human settlements, livestock farms, zooparks, Danube harbor). The rats were autopsied just after their arrival, and kidney and bladder samples were aseptically resected. The specimens were immediately analyzed or were kept in a −20 °C freezer before processing.

Main data on the animals—body size, weight, gender, site and time of capture/death—were recorded. Cell suspensions were made from kidney and urinary bladder tissues in sterile mortars with distilled water. Total tissue DNA was extracted from these suspensions with a Genomic DNA Mini Kit (Geneaid, Biotech Ltd., New Taipei City, Taiwan). A 1 µL sample from the eluted DNA was used as template in further PCR reactions. In 23 animals, urine was found in the bladders; these samples were screened for *Leptospirae* by dark-field microscope.

### 2.2. Collection and Preparation of Human Samples

Between 2022 and 2025, a total of 353 samples (165 urine; 5 tissues: 3 kidneys, 2 livers; 181 blood and 2 tracheal secretions) from 232 patients (72 females, 160 males) were submitted for *Leptospira* PCR testing to the National Center for Public Health and Pharmacy, which is the institute authorized to perform leptospirosis diagnostics with nationwide coverage. Thirty-nine patients were from the capital, Budapest, while 25 patients lived in the neighboring Pest County; the rest originated from other counties of Hungary. For most patients, their anamnestic data, exposure risk factors, and possible travel history were available. Some of the specimens were analyzed immediately after arrival, while the remaining samples were stored at −20 °C for two weeks. The human urine samples were screened for *Leptospirae* under a dark-field microscope after their arrival at the National Center for Public Health and Pharmacy.

DNA was isolated from tissues and from a portion of the urine samples using the High Pure DNA Template Preparation Kit (Roche Diagnostics GmbH, Mannheim, Germany). From anticoagulated blood samples, sera, and another portion of the urine samples, DNA extraction was performed using the QIAamp DNA Blood Mini Kit (QIAGEN GmbH, Hilden, Germany). A 9 µL sample from the eluted DNA was used as template in further PCR reactions. All the DNA specimens were kept at −20 °C.

### 2.3. PCR Assays

**Figure 1:**
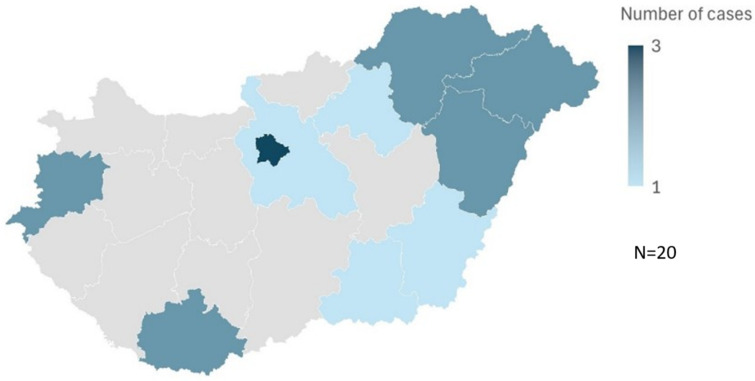
County distribution of *Leptospira*-positive human cases.

The DNA extracted from rat kidneys and bladders and from the human samples with suspected leptospirosis was tested using the BactoReal *Leptospira* spp. (16S + LipL32) multiplex kit (Ingenetix, Vienna, Austria). The positive samples were further studied by a single PCR assay [37] targeting a section of the lfb1 adhesin molecule coding gene. The distribution of residences of patients who were PCR-positive for *Leptospira* is shown in Figure 1.

In the PCR assays we used GoTaq G2 DNA Polymerase (Promega Corporation, Madison, WI, USA) and Promega dNTPs. The 20 μL reaction volume of the PCR mix consisted of 7.9 μL ddH_2_O, 4 μL of 5× colorless GoTaq reaction buffer, 0.5 μL of each primer (20 μM), 4 μL dNTP mix (1 mM), 0.1 μL GoTaq G2 polymerase (5 u/μL), and 3 μL of template DNA. We used the cycling parameters described by the developers of the assays. Finally, the amplicons of the PCR assays were visualized by agarose gel electrophoresis.

### 2.4. Dark-Field Microscopy

Urine was found in the urinary bladders of 23 of the sampled rats. Fresh urine samples (≤24 h) were examined by dark-field microscopy. The human urine samples were examined similarly after their arrival.

### 2.5. DNA Sequencing

The PCR products were isolated and purified using Exonuclease I (Applied Biosystems, Thermo Fisher Scientific, Waltham, MA, USA) and FastAP Thermosensitive Alkaline Phosphatase (Thermo Fisher Scientific) according to the manufacturer’s instructions. PCR amplification for Sanger sequencing was carried out using primers from the lfb1-specific PCRs, on both strands, applying the BigDyeTM 3.1v Terminator Cycle Sequencing Kit (Thermo Fisher Scientific). Purification of the target sequence was performed using the BigDye XTerminator Purification Kit (Thermo Fisher Scientific). Capillary electrophoresis was performed on an ABI 3500 Genetic Analyzer at the National Center for Public Health and Pharmacy.

### 2.6. Sequence Analysis

Sequence identity was confirmed through the Pasteur Institute’s BIGSdb database (version 1.52.2) using the BLAST plugin (version 1.7.1.) to query the sequences against selected isolate data (https://bigsdb.pasteur.fr/cgi-bin/bigsdb/bigsdb.pl?db=pubmlst_leptospira_isolates, accsessed on 10 October 2025). The obtained sequences were aligned with all lfb1 sequences available in the database.

The *Analysis* function of the *Leptospira* cgMLST database was used with the BLAST plugin. The query sequence was pasted into the database, and the following options were selected: *list all isolates*; among the search results, at least the species name and the lfb1 type were displayed. The search results were exported to Excel, and the resulting table was filtered for 100% sequence identity and maximum alignment length.

The obtained results were verified as follows. Reference sequences corresponding to the lfb1 types identified after Excel filtering were downloaded from the Pasteur database. We aligned our own sequences to the given *lfb1* reference sequence using MEGA11 software.

Each reference sequence was 278 bp in length. The Pasteur database currently distinguishes 78 different lfb1 types. The institutional database compares the submitted sequence with a well-defined 278 bp region of the lfb1 gene. The simplified lfb1-based typing scheme is based on single-nucleotide polymorphisms (SNPs) present within this 278 bp region. The genetic distances between the individual lfb1 types are summarized in Table 1. The validity of this approach was demonstrated in a study on the genetic diversity of *Leptospira* strains circulating in humans and dogs [38]. Therefore, the differentiation of *lfb1* types was not performed arbitrarily based on a self-defined number of SNPs; instead, our results were integrated into an established system.

## 3. Results

### 3.1. PCR of the Rat Samples

Out of 190 tested animals, identification of *Leptospira* genomes from the renal tissues was successful in 103 cases (54.2%) (Table S2). As sequencing of the PCR products revealed, all detected strains were a pathogenic species: *L. interrogans*, lfb1 species group 1.

### 3.2. PCR, Sequencing and Epidemiology of Human Samples

Among the patients presenting with suspected leptospirosis, pathogen DNA was detectable in 8% of the cases (Table S1). The low detection rate may also be attributed to antibiotic therapy initiated prior to sample collection. Twenty PCR products were of sufficient quality for sequencing, representing 14 individual patients. Among the 14 positive patients, *L. interrogans* was identified in 12 cases, and according to the lfb1 typing, they belonged to species group 2. In one Budapest resident, lfb1 species group 3 of *L. interrogans* was identified. This patient’s travel history included Bali, Indonesia; therefore, this case was most likely an imported one. In another patient, *L. kirschneri* belonging to lfb1 species group 6 was identified. Table 2 summarizes the numbers of our samples and the SNP differences between the identified *lfb1* types.

**Figure 2:**
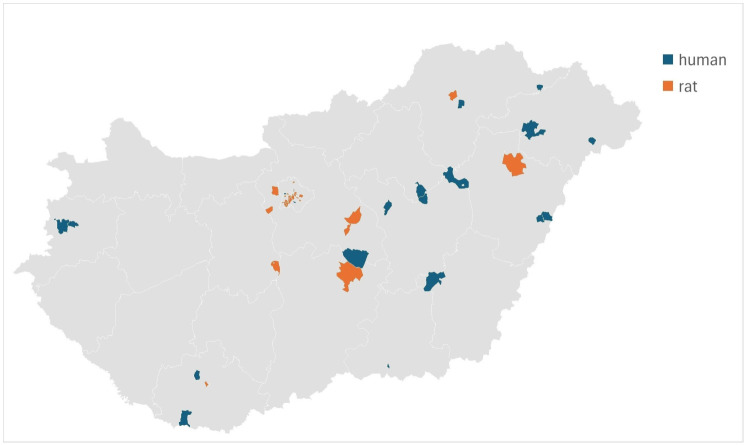
The national distribution of *Leptospira*-positive human and rat cases reported by zip code.

**Figure 3:**
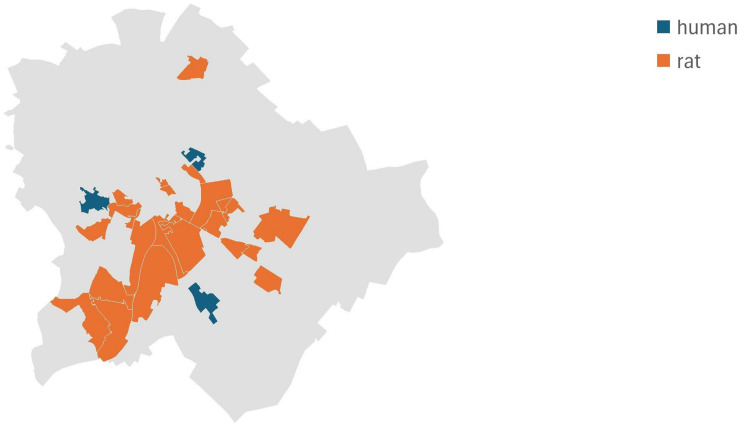
The distribution of *Leptospira*-positive human and rat cases in districts of Budapest reported by zip code.

The latter patient resides in the northeastern part of the country; their medical history is unknown. For eight patients, specific symptoms were observed; most commonly, impaired renal function, as well as liver failure and, in some cases, thrombocytopenia, was reported. In four cases, fever and muscle and joint pain were recorded. In eight cases, rodent contact was indicated on the submission form, while in three cases, risk factors were reported, including animal keeping, fishing, and poor socioeconomic conditions. For the geographical distribution of rats and human patients in Budapest and in the whole country reported by zip code, see Figure 2 and Figure 3.

### 3.3. Dark-Field Microscopy Studies

Dark-field microscopy analysis of the 23 rat urine samples and all human urine samples resulted in negative results; *Leptospira* line forms were not detectable.

As the applied methods, sources of samples, and results are quite complex, Table 3 summarizes the basis and some details of our findings.

## 4. Discussion

Our results indicate that a zoonotic bacterium is profoundly present in free-ranging rat populations of large and small towns, villages, and livestock farms in a Central European country. As Leptospires persist and establish chronic infections in renal tubuli and urinary tracts, free-ranging rats and their urine pose a constant epidemiological threat not only to our livestock animals and pets but also to humans. Leptospirosis is not just a tropical disease, since 54.2% of free-ranging rats in Hungary are chronically infected by pathogenic Leptospires, as this study shows. The occurrence of pathogenic Leptospires in rats is still a constant threat to humans, livestock and pets.

PCR and sequencing analysis of the rat samples revealed that only one lfb1 group circulates in the whole sampled Hungarian free-ranging rat population. Rat individuals probably maintain and spread their *Leptospira* infections to each other in their environments, isolated from humans and probably from other animal species. There is no information about the clinical signs or possible death toll among rat individuals, but the prevalence data on *Leptospira* infection in rats indicate that the infection is widespread and continuously maintained. Why *L. interrogans* lfb1 species group 1 is the prevalent *Leptospira* species group in the Hungarian rat population is not known. The lfb1 species group 1 adhesin molecule type probably effectively enables the bacteria to establish chronic infections in rats.

As far as the human samples are concerned, most infections were caused by *L. interrogans* species group 2. Based on the above, there are no identifiable infection hotspots; in most cases rodent contact can be established as the underlying factor. The cases are associated with poor socioeconomic conditions or with occupations (e.g., animal husbandry) and hobbies (e.g., fishing) that constitute risk factors.

Among neighboring countries, a surveillance study covering 410 patients was conducted in Slovenia. Via real-time PCR on samples from clinically suspected patients, the pathogen’s DNA was detected in 11.8% of these cases [38]. In Portugal (which is considered an endemic area), a 22.7% real-time PCR positivity rate was observed [39]. In similar studies researchers focused on cattle in Austria and on small mammals in Germany. The Austrian study identified the pathogen’s DNA in 1.2% of the screened cattle [40], while in Germany, the highest prevalence was measured in certain voles (*Microtus* spp.), where it reached 30% frequency [41].

Although we regularly emphasize the importance of molecular testing in Hungary, only about 10% of submitting clinicians consider sending anticoagulated blood and/or urine samples for such analyses. When leptospirosis is suspected, international protocols recommend the immediate initiation of antibiotic therapy, which in many cases precedes sample collection by several days. Considering all these factors, the above-mentioned 8% PCR positivity rate can even be regarded as relatively high. The species and species group diversity could not be assessed due to the low number of PCR-positive human cases.

In Hungary, 0.05 cases per 100,000 population were reported in 2023, according to data from the European Centre for Disease Prevention and Control (ECDC). Slovakia shows a similar positivity rate, whereas the notification rate of human leptospirosis is 0.58 per 100,000 in Romania and 1.32 per 100,000 in Slovenia. The average notification rate in the European Union is 0.3 per 100,000 population.

In the majority of human cases, confirmation of infection is based exclusively on serological methods; therefore, reliable data on PCR positivity rates are not available.

As far as the rat–human *Leptospira* relationship is concerned, (similarly to a publication from Canada [29]) we could not detect any direct associations between rat and human *Leptospirae*; i.e., at least in Hungary, in symptomatic human cases, rats are not the sources of infection. Rats probably infect each other with lfb1 species group 1 of *Leptospira interrogans*, maintaining isolated circulation of infection inside their populations. Although *Leptospira* species are ubiquitous in rat populations, human disease is most common in the tropics [1], particularly in Southeast Asia, Oceania, the Indian subcontinent, the Caribbean and Latin America [2], which indicates that some climate, temperature, and social factors are necessary for more efficient rat–human *Leptospira* transmission.

In the human cases, we could not identify any epidemics; only individual infections happened, probably with various infective sources and *Leptospira* types (*L. interrogans* species groups 2 and 3, *L. kirschneri* species group 6). As the main message of this paper, we found proof that, despite their large populations living in close proximity to humans and their high *Leptospira* prevalence levels, at least in symptomatic cases of human *Leptospira* infections in Hungary, rats cannot be considered an important vector or source of human *Leptospira* infections. This is probably not true for all countries of the world. The density of rat and human populations, their proximity or isolation, hygiene and climate circumstances, and many other factors can influence the spread and maintenance of *Leptospira* infections and the ability of the bacteria to be infectious for other species.

The limitations of our work include the low number of human patients, but we could not influence that as in Hungary (and Europe) human leptospirosis is a rare, sporadic infection with low case numbers. We did not know where and when the human patients were infected; they were (as in most epidemiological studies) registered by their official home addresses. That is why it is not a problem that the sites of rat capture did not coincide with the sites of human infections (which we do not know).

The question of what animals (if not rats) and factors could be sources of human *Leptospira* infections in Europe remains unanswered. Continuous improvements of the sanitary systems and hygienic behavior in developed countries mitigate the possibility of direct contact with rats. However, changes in outdoor leisure activities open new fields for human infections. For instance, hunting is increasingly popular, which provides a new possible route of infection, as hunters dissect the bodies on the spot. *Leptospira* infection was shown in 6% of red foxes (*Vulpes vulpes*) and 38% of wild bores (*Sus scrofa*) in France [42], but sensitivity of red deer and Mustelid species was also reported from New Zealand [43]. Human infections were identified among triathlon competitors swimming in surface waters [44]. Less frequent but heavier rainfalls and floods also increase the possibility of *Leptospira* contamination. Rats are not the only rodent species that could carry and spread *Leptospirae*. In China, *Leptospirae* were isolated [45] from eight non-Rattus rodent species, with the house mouse (*Mus musculus*) and striped field mouse (*Apodemus agrarius*) among them; these are also widespread rodent species in Europe. House mice live in large numbers, in very close proximity to (or in) human houses and flats, while striped field mice are frequent in meadows, farms, and forests in Europe. Imported cases also continuously occur, particularly among travelers engaged in adventure tourism and water-related activities in endemic countries of the tropics. In the Netherlands, 14% of the total human *Leptospira* cases over a four-year-long period were imported [46].

A large-scale survey study on house mice, pets (hamsters), livestock (mostly cattle) and companion animals (cats, dogs) would be beneficial to identify the exact sources of human *Leptospira* infections in Hungary.

## 5. Conclusions

The dominant presence of the *L. interrogans* lfb1 species group 1 type of *Leptospirae* in rats and *L. interrogans* lfb1 species group 2 in humans indicates that there is no direct association between human and rat *Leptospira* infections in Hungary. The sources of the sporadic human infections were probably other rodents or livestock, or pet/game animals. This is not surely true for all countries, where hygienic and sanitary circumstances profoundly influence possible rat–human epidemiological associations. Further diagnostic works are needed to collect data on whether our findings are generally true in developed countries, while rats are still the main sources of human *Leptospira* infections in the developing countries of the tropics.

## Figures and Tables

**Table 1:** Pairwise distances of lfb1 genotypes.

Pairwise Distance (Mega)			
	*L._interrogans*_lfb1: 1	*L._interrogans*_lfb1: 2	*L._interrogans*_lfb1: 3
*L._interrogans*_lfb1: 1			
*L._interrogans*_lfb1: 2	0.01092		
*L._interrogans*_lfb1: 3	0.01091	0.00724	
*L._kirschneri*_lfb1: 6	0.05671	0.06890	0.06880

**Table 2:** Sequence differences between lfb1 genotypes in our isolates.

	Number of Our Samples																																							
*L. interrogans* lfb1: 1	103 (rats)	A	T	T	C	T	T	C	C	T	C	G	C	C	C	T	T	C	T	A	G	T	A	T	T	T	G	G	T	T	T	T	A	T	T	T	C	A	A	C
*L. interrogans* lfb1: 2	12 (human)	.	.	.	.	.	.	.	.	.	.	.	.	.	.	.	.	.	.	.	.	.	.	.	.	.	.	.	.	.	.	.	.	.	.	.	.	.	.	.
*L. interrogans* lfb1: 3	1 (human, imported)	.	.	.	.	.	.	.	.	.	.	.	.	.	.	.	.	.	.	.	.	.	.	.	.	.	.	.	.	.	.	.	.	.	.	.	.	.	.	.
*L. kirschneri* lfb1: 6	1 (human)	.	.	.	.	.	.	.	.	.	.	.	.	.	.	.	.	.	.	.	.	.	.	.	.	C	.	.	.	.	.	.	.	.	.	.	.	G	.	.
																																								78
*L. interrogans* lfb1: 1	103 (rats)	C	**G**	C	T	T	A	C	G	C	A	C	A	G	A	T	C	G	G	T	C	A	A	A	T	C	A	A	T	C	C	T	T	C	C	T	C	C	A	T
*L. interrogans* lfb1: 2	12 (human)	.	**T**	.	.	.	.	.	.	.	.	.	.	.	.	.	.	.	.	.	.	.	.	.	.	.	.	.	.	.	.	.	.	.	.	.	.	.	.	.
*L. interrogans* lfb1: 3	1 (human, imported)	.	T	.	.	.	.	.	.	.	.	.	.	.	.	.	.	.	.	.	.	.	.	.	.	.	.	.	.	.	.	.	.	.	.	.	.	.	.	.
*L. kirschneri* lfb1: 6	1 (human)	.	.	.	.	.	.	.	.	.	.	.	.	.	.	.	.	.	.	.	.	.	.	.	.	.	.	.	.	.	.	.	.	.	T	.	.	.	.	.
*L. interrogans* lfb1: 1	103 (rats)	T	A	G	C	G	G	T	A	A	A	T	A	C	A	A	G	G	T	T	T	C	T	G	G	A	A	C	C	A	A	C	C	C	A	A	A	C	G	G
*L. interrogans* lfb1: 2	12 (human)	.	.	.	.	.	.	.	.	.	.	.	.	.	.	.	.	.	.	.	.	.	.	.	.	.	.	.	.	.	.	.	.	.	.	.	.	.	.	.
*L. interrogans* lfb1: 3	1 (human, imported)	.	.	.	.	.	.	.	.	.	G	.	.	.	.	.	.	.	.	.	.	.	.	.	.	.	.	.	.	.	.	.	.	.	.	.	.	.	.	.
*L. kirschneri* lfb1: 6	1 (human)	C	.	.	.	.	.	.	.	.	.	.	.	.	.	.	.	.	.	.	.	.	.	.	.	.	.	.	.	.	.	.	.	.	G	.	.	.	.	.
																																								156
*L. interrogans* lfb1: 1	103 (rats)	T	T	C	C	T	C	T	T	A	C	A	A	C	G	G	T	A	G	C	G	T	T	A	C	G	A	T	C	T	C	T	C	A	A	T	C	T	A	A
*L. interrogans* lfb1: 2	12 (human)	.	.	.	.	.	.	.	.	.	.	.	.	.	.	.	.	.	.	.	.	.	.	.	.	.	.	.	.	.	.	.	.	.	.	.	.	.	.	.
*L. interrogans* lfb1: 3	1 (human, imported)	.	.	.	.	.	.	.	.	.	.	.	.	.	.	.	.	.	.	.	.	.	.	.	.	.	.	.	.	.	.	.	.	.	.	.	.	.	.	.
*L. kirschneri* lfb1: 6	1 (human)	.	.	.	.	.	.	.	.	.	.	G	G	.	.	.	.	.	.	.	.	.	.	.	.	.	.	.	T	.	.	.	G	.	.	.	.	.	.	.
*L. interrogans* lfb1: 1	103 (rats)	C	G	G	A	G	A	A	T	A	C	C	T	T	T	T	T	A	C	C	T	G	G	A	C	G	G	T	**C**	G	C	T	G	G	T	C	A	A	A	C
*L. interrogans* lfb1: 2	12 (human)	.	.	.	.	.	.	.	.	.	.	.	.	.	.	.	.	.	.	.	.	.	.	.	.	.	.	.	**T**	.	.	.	.	.	.	.	.	.	.	.
*L. interrogans* lfb1: 3	1 (human, imported)	.	.	.	.	.	.	.	.	.	.	.	.	.	.	.	.	.	.	.	.	.	.	.	.	.	.	.	.	.	.	.	.	.	.	.	.	.	.	.
*L. kirschneri* lfb1: 6	1 (human)	.	.	.	.	.	.	.	.	.	.	.	.	.	.	.	.	.	.	.	.	.	.	.	.	.	.	.	.	.	.	C	.	.	.	.	.	.	.	.
																																								234
*L. interrogans* lfb1: 1	103 (rats)	T	T	T	C	A	C	A	G	G	A	A	C	C	G	G	A	A	C	C	C	T	T	G	A	A	G	G	T	A	C	T	A	C	T	T	T	G	A	C
*L. interrogans* lfb1: 2	12 (human)	.	.	.	.	.	.	.	.	.	.	.	.	.	.	.	.	.	.	.	.	.	.	.	.	.	.	.	.	.	.	.	.	.	.	.	.	.	.	.
*L. interrogans* lfb1: 3	1 (human, imported)	.	.	.	.	.	.	.	.	.	.	.	.	.	.	.	.	.	.	.	.	.	.	.	.	.	.	.	.	.	.	.	.	.	.	.	.	.	.	.
*L. kirschneri* lfb1: 6	1 (human)	.	.	.	.	.	.	.	.	.	.	.	.	.	.	.	.	.	.	.	.	.	.	.	.	.	.	.	.	.	.	.	.	.	.	.	.	.	.	.
*L. interrogans* lfb1: 1	103 (rats)	A	G	T	A	G	A	T	T	G	G	G	G	A	G	A	A	A	C	A	G	A	A	C	C	**G**	G	T	A	A	T	C	T	A	T	G	A	A	G	T
*L. interrogans* lfb1: 2	12 (human)	.	.	.	.	.	.	.	.	.	.	.	.	.	.	.	.	.	.	.	.	.	.	.	.	**A**	.	.	.	.	.	.	.	.	.	.	.	.	.	.
*L. interrogans* lfb1: 3	1 (human, imported)	.	.	.	.	.	.	.	.	.	.	.	.	.	.	.	.	.	.	.	.	.	.	.	.	A	.	.	.	.	.	.	.	.	.	.	.	.	.	.
*L. kirschneri* lfb1: 6	1 (human)	.	.	.	.	.	.	.	.	.	.	.	.	.	.	.	G	G	T	T	.	.	.	.	.	.	.	.	G	.	.	.	.	.	.	.	.	.	.	.
																																								278
*L. interrogans* lfb1: 1	103 (rats)	A	A	A	A	A																																		
*L. interrogans* lfb1: 2	12 (human)	.	.	.	.	.																																		
*L. interrogans* lfb1: 3	1 (human, imported)	.	.	.	.	.																																		
*L. kirschneri* lfb1: 6	1 (human)	.	.	.	.	.																																		

**Table 3:** Summary of sample types, applied methods and results for rat and human specimens.

Sample Types	Rat Samples	Human Samples
Urine (dark-field microscopy negative)	23	165
Tissue	190	5
Blood	-	181
Tracheal secretion	-	2
Samples analyzed by commercial PCR kit	190	353
PCR-positive samples	103	29
Samples successfully sequenced	103	20 samples (14 patients)
lfb1 genotype distribution	lfb1 type 1: 103	lfb1 type 2: 12 patients
		lfb1 type 3: 1 patient
		lfb1 type 6: 1 patient

## Data Availability

All datasets generated and used for this study are available in the main text or provided by the corresponding author upon request.

## Supplementary Materials

The following supporting information can be downloaded at: https://www.mdpi.com/article/10.3390/pathogens15070729/s1, Table S1: Table of human samples; Table S2: RAT samples.
